# Temporary Relative Lumbar Spinal Stenosis After Skip Partial Laminectomies in an Elderly Woman: A Case Report

**DOI:** 10.7759/cureus.91003

**Published:** 2025-08-26

**Authors:** Hayato Kinoshita, Michio Hongo, Hiroshi Matsuura, Masaaki Takeshima, Naohisa Miyakoshi

**Affiliations:** 1 Orthopaedics, Honjo Daiichi Hospital, Yurihonjo, JPN; 2 Physical Therapy, Akita University Graduate School of Medicine, Akita, JPN; 3 Orthopaedic Surgery, Akita University Graduate School of Medicine, Akita, JPN

**Keywords:** bernoulli’s principle, lumbar spinal stenosis (lss), partial laminectomy, transient paraparesis, venturi effect

## Abstract

Lumbar spinal stenosis is common and mostly caused by age-related spine degeneration. When conservative treatment is not successful, surgery is typically performed. We encountered a 79-year-old female patient who developed spinal canal stenosis at an unoperated level after partial skip laminectomies at multiple lumbar levels. On postoperative day two, she developed paraparesis, and magnetic resonance imaging showed lumbar spinal canal stenosis at a level that was normal before surgery. Her paraparesis resolved two weeks after surgery. Repeat imaging at the three-month follow-up showed an improvement in the lumbar stenosis. We consider this phenomenon to be "negative pressure concentration in the dural canal" based on the Venturi effect and Bernoulli's principle.

## Introduction

Lumbar spinal stenosis (LSS) is common and mostly caused by age-related spine degeneration. The typical clinical manifestation is intermittent neurogenic claudication, accompanied by paresthesia in the lower limbs, which reduces the ability to walk [1-3]. Treatment can be broadly divided into conservative and surgical options. Conservative treatments include lifestyle modification, lumbar epidural steroid injections, nerve root block, physiotherapy, and medication [4-6]. If surgery is elected, the selection of the procedure is mainly determined by anatomical issues. A partial or complete laminectomy, with or without foraminotomy, may be performed as indicated, depending on the number of levels involved, the laterality of the pathology and symptoms, and the location of the stenosis (central or foraminal). Fusion with or without fixation may be added if kyphosis, scoliosis, or spondylolisthesis is present. The surgeon's preference and experience are also factors. The superiority of conservative or surgical treatment is still a matter of debate, but in patients with severe neurological symptoms, surgical treatment tends to be the first choice [7,8].

Lumbar spine surgery is associated with potential complications, which include epidural hematoma, cerebrospinal fluid (CSF) leak, deep venous thrombosis, pulmonary embolism, pneumonia, urinary tract infection, and wound infection [9]. In particular, nerve root compression from an epidural hematoma can result in serious sequelae such as irreversible paraplegia and dysfunction of the bladder and/or bowel. We report the case of a woman who developed temporary paraparesis after undergoing partial laminectomies for LSS skip lesions. The postoperative magnetic resonance imaging (MRI) revealed a narrowing of the dural canal in the non-operated area. Consequently, we examined the literature and considered the findings.

## Case presentation

A 79-year-old woman presented with a two-year history of numbness in her lower limbs, right calf pain, and associated gait disturbance. Three months previously, her symptoms had worsened, so she requested surgical treatment. On physical examination, she exhibited decreased reflexes in the patellar and Achilles tendons and decreased superficial sensation along the lateral aspect of both legs. Manual muscle testing (MMT) in the lower limbs was normal.

**Figure 1:**
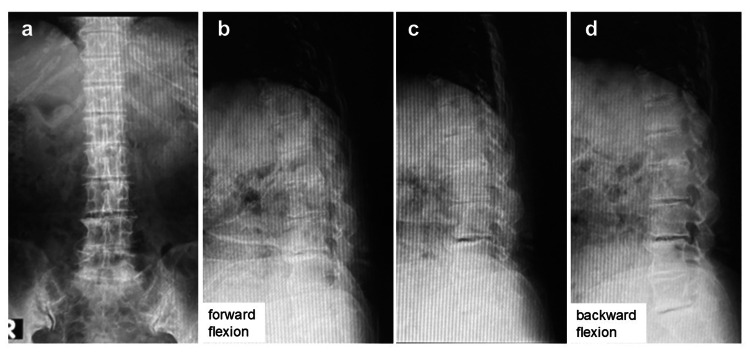
Preoperative X-ray Preoperative X-ray showing lumbar degeneration and mild kyphosis, not showing lumbar spondylolisthesis. (a) Anteroposterior view; (b) forward flexion; (c) neutral position; (d) backward flexion.

Her preoperative Japanese Orthopaedic Association and visual analog pain scores were 13 (out of 29) and 6 (out of 10), respectively. Plain radiography showed lumbar degeneration and mild lumbar kyphosis but no lumbar spondylolisthesis (Figure 1).

**Figure 2:**
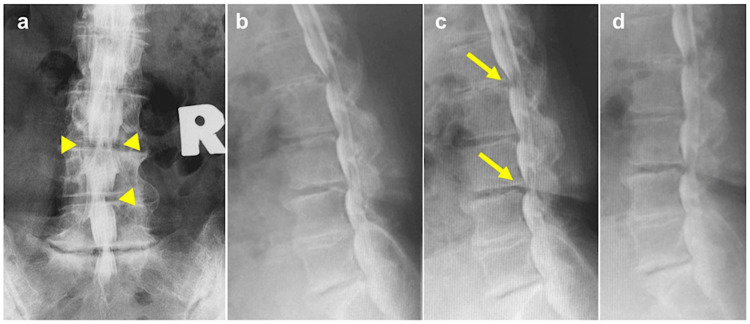
Preoperative myelography (a) Anteroposterior view. L3/4 hourglass stenosis, L4/5 right side large defect of dural sac (yellow arrowheads); (b) forward flexion; (c) neutral position. Moderate anterior indentation of L1/2 and L3/4 (yellow arrows); (d) backward flexion.

Myelography showed L3/4 hourglass stenosis, a large contrast defect at L4/5 on the right, and moderate anterior indentation at L1/2 and L3/4 (Figure 2).

**Figure 3:**
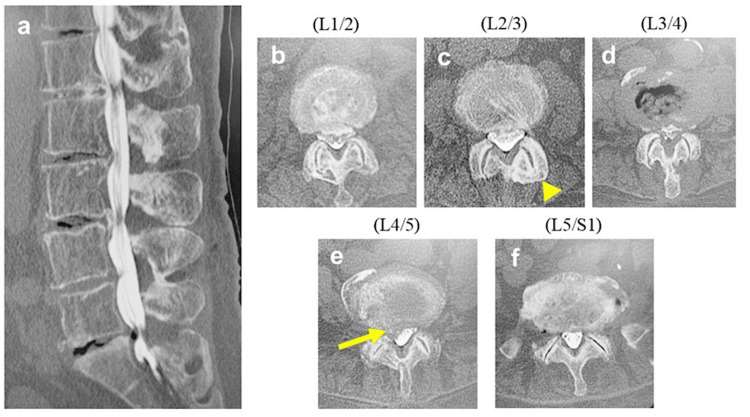
Preoperative CT myelography (a) Sagittal image of lumbar spine. (b) Axial image of L1/2. Lumbar spinal canal stenosis was shown. (c) Axial image of L2/3. The L2/3 facet with partial union was shown (yellow arrowhead). (d) Axial image of L3/4. Lumbar spinal canal stenosis was shown. (e) Axial image of L4/5. L4/5 calcificated herniation was shown (yellow arrow). (f) Axial image of L5/S1.

Computed tomography myelography showed an L4/5 calcified disc herniation, a partially fused left L2/3 facet joint, and L1/2 and L3/4 stenosis (Figure 3).

**Figure 4:**
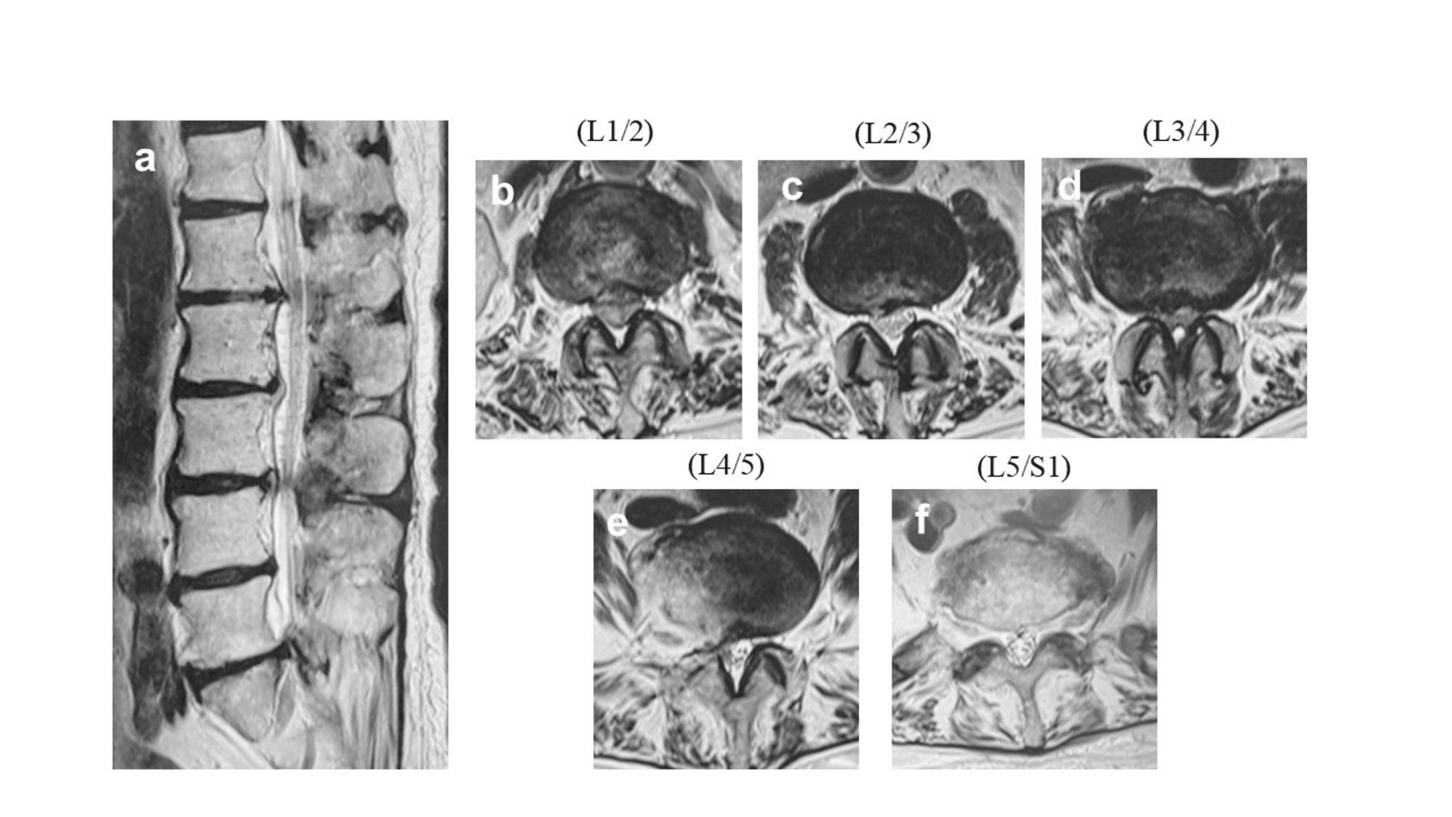
Preoperative MRI (a) Sagittal image of lumbar spine. (b) Axial image of L1/2. Lumbar spinal canal stenosis was shown. (c) Axial image of L2/3. (d) Axial image of L3/4. Lumbar spinal canal stenosis was shown. (e) Axial image of L4/5. Lumbar spinal canal stenosis was shown on the right side of L4/5. (f) Axial image of L5/S1.

MRI revealed stenosis at L1/2 and L3/4, as well as right L4/5 stenosis (Figure 4).

**Figure 5:**
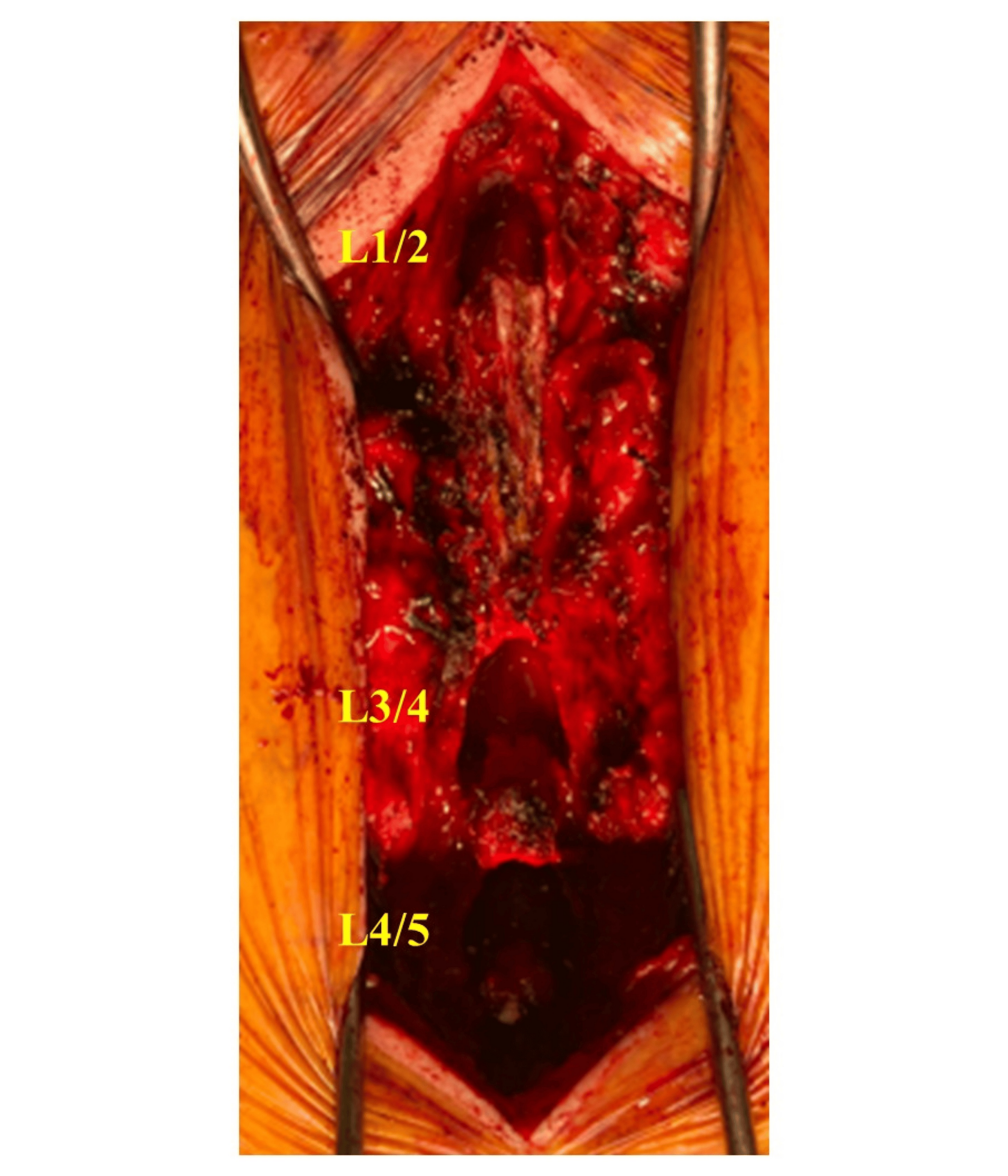
Operative findings Operative findings: conducting L1/2, L3/4, and L4/5 partial laminectomy.

The patient underwent partial laminectomies at L1/2, L3/4, and L4/5 without intraoperative complications (Figure 5). The posterior approach was performed by longitudinally splitting the spinous processes from L1 to L4. Bone resection on the spinous process side was performed until the chevron of the yellow ligament was visible. The yellow ligament was split vertically in the middle, separated into left and right sides, and then removed. The operative time was two hours and 23 minutes, and bleeding was 206 ml. The postoperative drain output was 30 ml.

**Figure 6:**
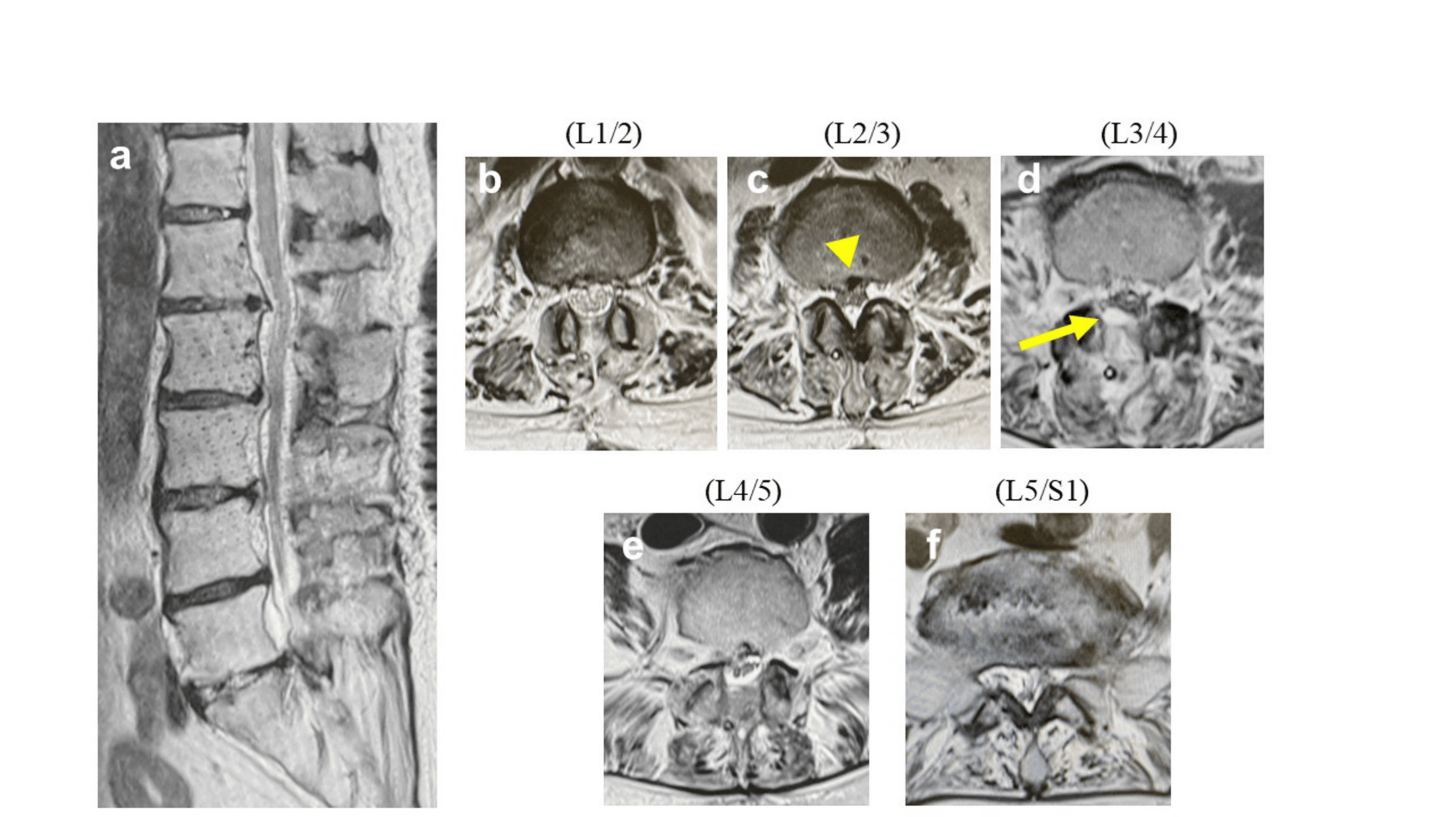
MRI on the second day after surgery (a) Sagittal image of lumbar spine. The spinal canal was decompressed at L1/2, L3/4, and L4/5. (b) Axial image of L1/2. (c) Axial image of L2/3. Cauda equina consolidation at the L2/3 level was shown (yellow arrowhead), which was not operated on. (d) An axial image of L3/4. The right-leaning epidural hematoma of L3/4 was shown (yellow arrow). (e) Axial image of L4/5. (f) Axial image of L5/S1.

The day after surgery, her numbness and leg pain had improved. On the second day, she developed dull pain and mild sensory impairment in the anterior left thigh and muscle weakness in the left lower extremity (MMT scores were 2 in the iliopsoas and 4 in the quadriceps). Lumbar spine MRI at the time showed a predominantly right-sided epidural hematoma at L3/4 and consolidation of the cauda equina nerve roots at L2/3, a level at which we did not operate (Figure 6). Postoperative MRI showed that decompression of the spinal canal was sufficient, and the hematoma at L3/4 was also compressing the right side of the spinal canal rather than the left side, with symptoms. Therefore, emergency surgery was deemed unnecessary, and the patient was observed for the rest of the day.

**Figure 7:**
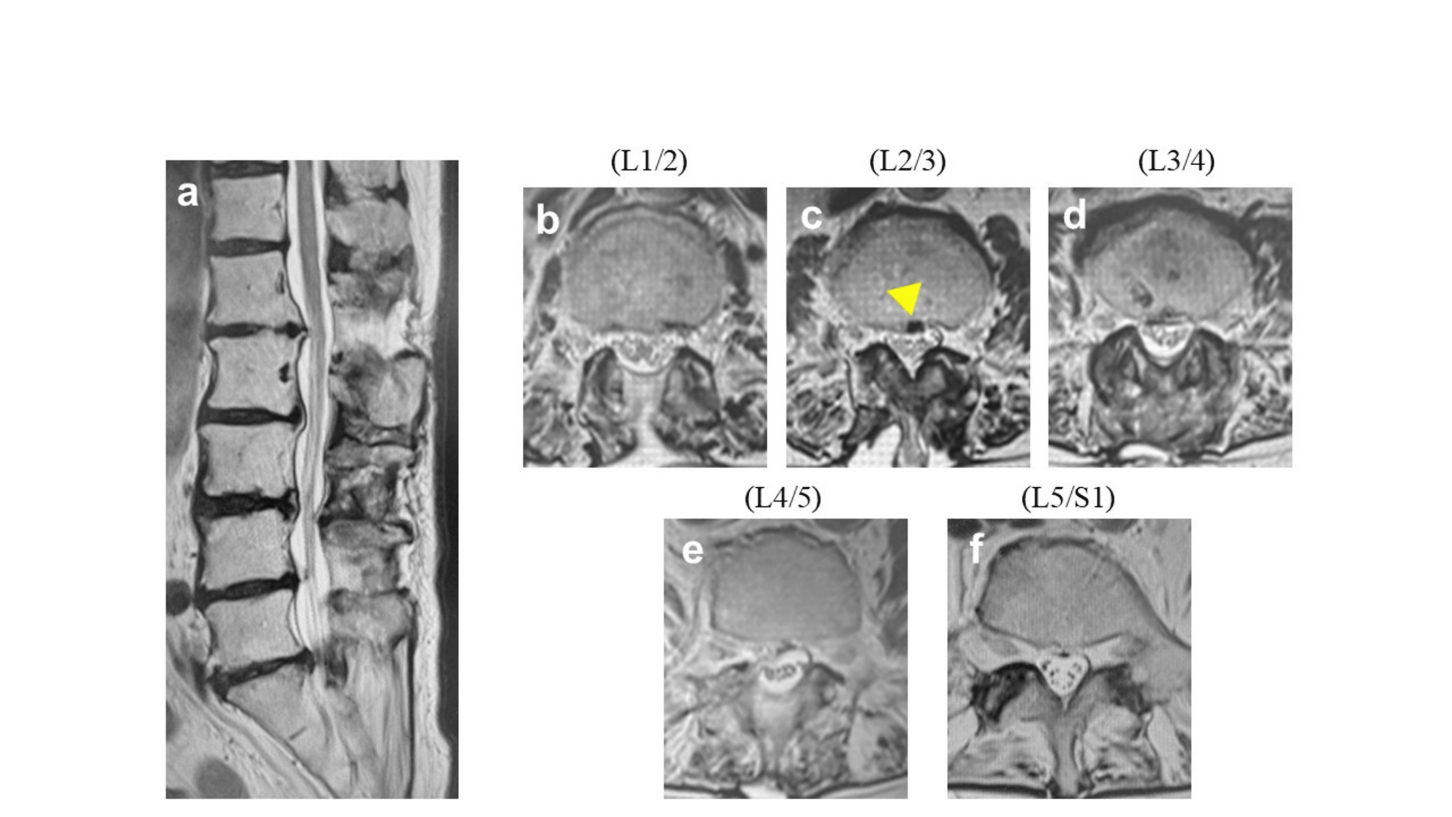
MRI at three months after surgery (a) Sagittal image of the lumbar spine. (b) Axial image of L1/2. (c) Axial image of L2/3. Cauda equina consolidation was improved (yellow arrowhead). (d) Axial image of L3/4. The right-leaning epidural hematoma of L3/4 had disappeared. (e) Axial image of L4/5. (f) Axial image of L5/S1.

The drain was removed on postoperative day three because of an improvement in muscle strength (MMT scores were 3 in the iliopsoas and 5 in the quadriceps). Four days later, the left anterior thigh pain had improved, and the left iliopsoas MMT score improved to 4. Four weeks after surgery, the patient was discharged from the hospital with the ability to walk with the assistance of a T-cane, although she still required supervision. The Japanese Orthopaedic Association score at discharge was 20 (improved from 13), and the visual analog pain score was zero. At the three-month follow-up, her left iliopsoas MMT score improved to 5, and she was able to walk independently with a T-cane; MRI showed improvement in the L2/3 spinal canal stenosis (Figure 7).

**Figure 8:**
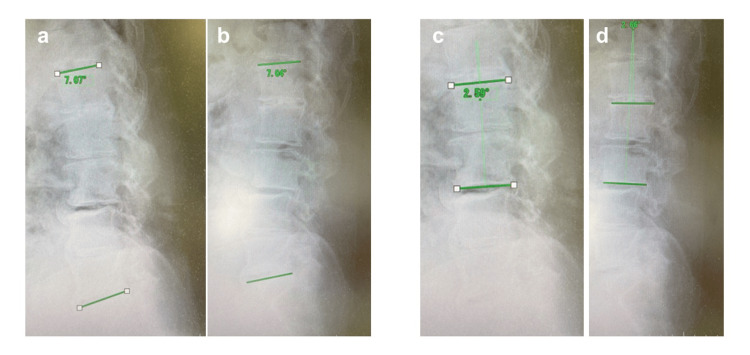
Pre- and postoperative lumbar lordosis and the local kyphosis angle (a) Preoperative lumbar lordosis. (b) Postoperative lumbar lordosis. Both angles were the same at 7 degrees. (c) Preoperative local kyphosis angle. (d) Postoperative local kyphosis angle. Both angles were the same at 2.5 degrees. There was no difference before and after the surgery.

As part of the postoperative evaluation, the lumbar lordosis and the local kyphosis angle were measured both pre- and postoperatively (Figure 8).

**Figure 9:**
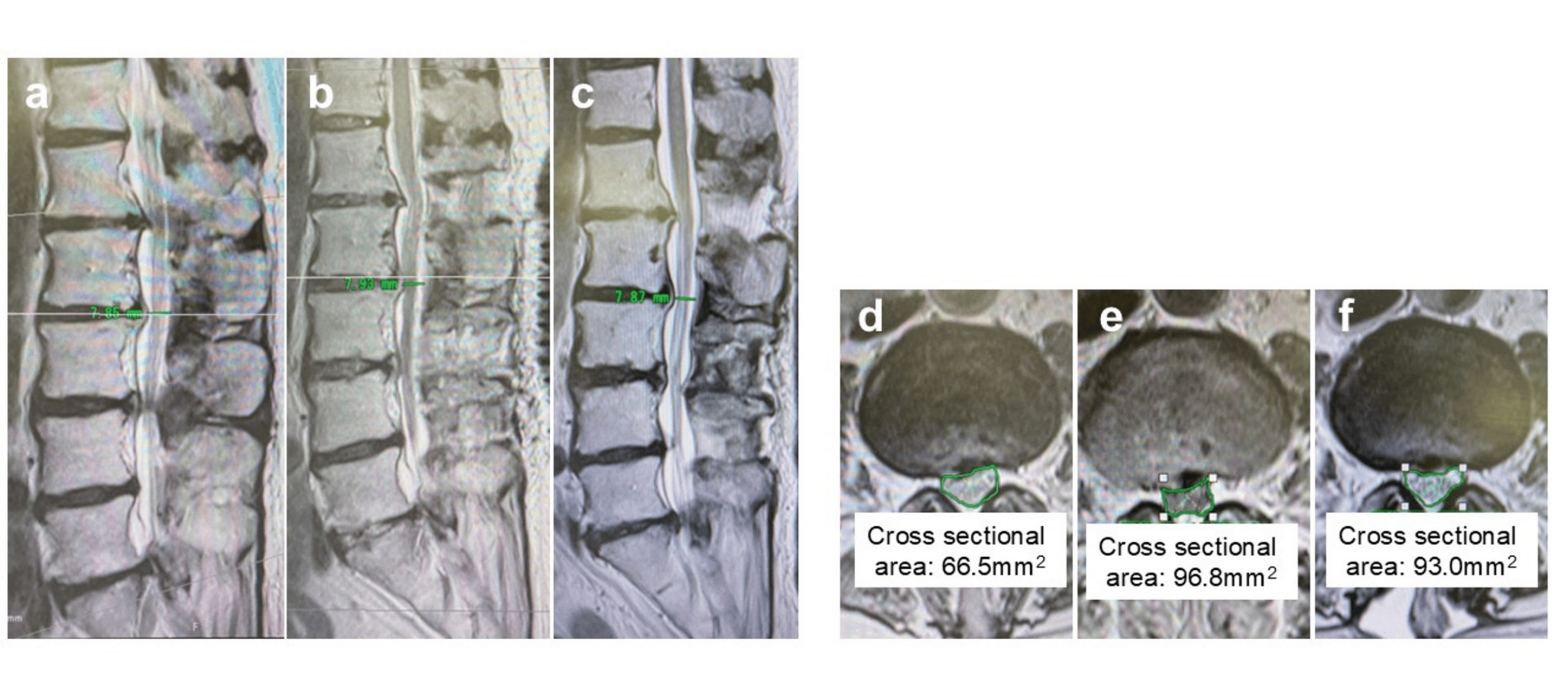
The anteroposterior diameter of the spinal canal and the dural canal cross-sectional area in the L2/3 level on MRI (a–c) The anteroposterior diameter of the spinal canal at the L2/3 level on MRI before surgery, immediately after, and at the three-month follow-up (all were 7.9 mm). (d–f) The cross-sectional area of the spinal canal at L2/3 on MRI before surgery, immediately after, and at the three-month follow-up (66.5 mm^2^ vs. 96.8 mm^2^ vs. 93.0 mm^2^, respectively).

The anteroposterior diameter of the spinal canal and the dural canal cross-sectional area (CSA) in the L2/3 level on MRI were also measured (Figure 9).

## Discussion

Kido et al. reported that weakness of the iliopsoas occurs mainly because of L2 and L3 nerve root disturbance [10]. Based on past literature, Lee et al. have shown that the skin segment on the anterior thigh corresponds to the L2 or L3 regions [11]. Therefore, the cause of our patient's serious left iliopsoas weakness and mild sensory impairment in the anterior left thigh may have been stenosis at L2/3 rather than the L3/4 epidural hematoma, because there was no foraminal stenosis at L3/4. We also considered that a change in lumbar alignment may have affected the spinal canal. In a cadaveric lumbar MRI study, Takiguchi et al. reported that the cauda equina moves ventrally and is exposed to tension when the lumbar spine is kyphotic [12]; in other words, lumbar kyphosis may exacerbate the symptoms of LSS. However, our patient's lumbar lordosis and local kyphosis angles remained at 7° and 2.5°, respectively, both before and after surgery, indicating no change (Figure 8).

The possibility of stenosis due to spinal instability was also considered, but it was thought to be unlikely because preoperative radiographs showed no spondylolisthesis and computed tomography scans revealed partial fusion of the left L2/3 facet joint.

Interestingly, when measuring the anteroposterior diameter and CSA of the spinal canal at L2/3 on MRI before surgery, immediately after, and at the three-month follow-up, we found that the CSA was lower after surgery (66.5 mm²) than before (96.8 mm²) and three months after (93.0 mm²). However, the anteroposterior diameter remained unchanged at all three time points (7.9 mm) (Figure 9).

Spinal subdural extra-arachnoid hygroma (SSEH) is listed as a differential diagnosis for this case. SSEH is similar to this case in that neurological symptoms in the lower limbs occur a few days after surgery. In addition, MRI reveals cauda equina consolidation [13]. However, in SSEH, CSF accumulates subdurally, so the postoperative narrowing of the dural canal shown in Figure 9 would not be observed; rather, it should become wider.

To the best of our knowledge, the narrowing of the dural canal immediately after surgery at a non-surgical site has not been previously reported. We hypothesize that the Venturi effect, based on Bernoulli's principle, affected our patient's spinal canal. The Venturi effect is a phenomenon that describes how the pressure of a fluid decreases as its velocity increases when it flows into a pipe constriction [14,15]. Bernoulli's principle is based on the law of conservation of energy applied to the flow of fluids; therefore, the opposite phenomenon of the Venturi effect occurs at the point of pipe expansion, where the fluid pressure increases and the flow slows down.

**Figure 10:**
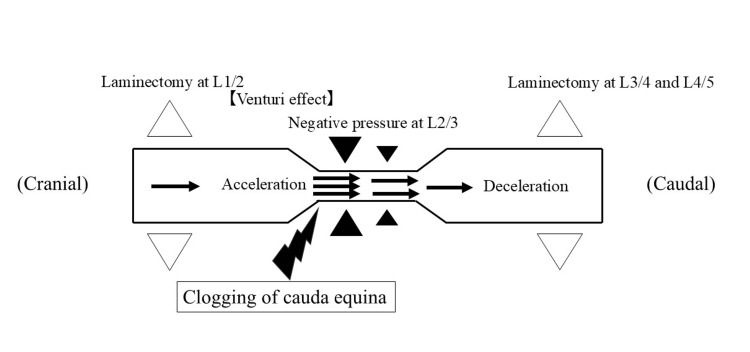
Our hypothesis based on the Venturi effect The Venturi effect may occur at the site of relative stenosis in the L2/3 level, which accelerates the flow of cerebrospinal fluid, resulting in decompression in the L2/3 dural canal. Next, the cerebrospinal fluid pressure at the caudal portion of the L2/3 level would have increased, and the flow would have slowed; the flow around the cauda equina would also have been slower, causing clogging and contributing to further narrowing of the dural canal. Image Credit: Hayato Kinoshita

In our patient, the widening of the dural canal in the area where the partial laminectomies were performed (L1/2, L3/4, and L4/5) may have resulted in relative stenosis at the non-surgical level (L2/3). The Venturi effect may have occurred at the cranial portion of L2/3, where the flow of CSF accelerated in an attempt to reduce the increased intramedullary pressure. Because the dura mater encasing the CSF is soft, it is possible that the dura there was then pulled inward by negative pressure, which reduced the dural canal CSA and caused transient LSS. At the caudal portion of L2/3, the CSF pressure would have increased and the flow slowed; the flow around the cauda equina would also have been slower, causing clogging and contributing to further narrowing of the dural canal (Figure 10).

If our hypothesis is correct, we should have also performed a laminectomy at L2/3 to have avoided creating relative stenosis, even though it may seem somewhat irrational. Minimally invasive laminectomy would be expected to cause less relative stenosis than conventional open laminectomy because there is less bone resection and soft tissue removal [16]. In addition, it may result in less excessive enlargement of the dural canal compared to conventional laminectomy and may reduce transient stenosis after skip surgery.

We have a limitation. As this is a single case report, it is not possible to determine whether stenotic lesions occurring in non-operative areas could cause neurological symptoms. Further studies are warranted to determine whether skip partial laminectomy for LSS produces transient stenosis because of the Venturi effect.

## Conclusions

This report has described a patient with LSS skip lesions who developed temporary relative stenosis at the normal level, which caused paraparesis after a skip laminectomy. Her symptoms improved two weeks after surgery. An MRI three months after surgery showed spontaneous resolution of the LSS at the normal level. We hypothesized that relative stenosis at the non-surgical level resulted in negative pressure within the dural sac at this level, owing to the Venturi effect and Bernoulli's principle, which temporarily exacerbated the LSS.
